# Regulation of CEACAM1 transcription in human breast epithelial cells

**DOI:** 10.1186/1471-2199-11-79

**Published:** 2010-11-04

**Authors:** Marieta Gencheva, Charng-Jui Chen, Tung Nguyen, John E Shively

**Affiliations:** 1 Department of Immunology, Beckman Research Institute of City of Hope, Duarte, CA 91010, USA

## Abstract

**Background:**

Carcinoembryonic antigen cell adhesion molecule 1 (CEACAM1) is a transmembrane protein with multiple functions in different cell types. CEACAM1 expression is frequently mis-regulated in cancer, with down-regulation reported in several tumors of epithelial origin and *de novo *expression of CEACAM1 in lung cancer and malignant melanoma. In this report we analyzed the regulation of CEACAM1 expression in three breast cancer cell lines that varied in CEACAM1 expression from none (MCF7) to moderate (MDA-MB-468) to high (MCF10A, comparable to normal breast).

**Results:**

Using *in vivo *footprinting and chromatin immunoprecipitation experiments we show that the *CEACAM1 *proximal promoter in breast cells is bound in its active state by SP1, USF1/USF2, and IRF1/2. When down-regulated the *CEACAM1 *promoter remains accessible to USF2 and partially accessible to USF1. Interferon-γ up-regulates CEACAM1 mRNA by a mechanism involving further induction of IRF-1 and USF1 binding at the promoter. As predicted by this analysis, silencing of IRF1 and USF1 but not USF2 by RNAi resulted in a significant decrease in CEACAM1 protein expression in MDA-MB-468 cells. The inactive *CEACAM1 *promoter in MCF7 cells exhibits decreased histone acetylation at the promoter region, with no evidence of H3K9 or H3K27 trimethylation, histone modifications often linked to condensed chromatin structure.

**Conclusions:**

Our data suggest that transcription activators USF1 and IRF1 interact to modulate CEACAM1 expression and that the chromatin structure of the promoter is likely maintained in a poised state that can promote rapid induction under appropriate conditions.

## Background

Carcinoembryonic antigen (CEA)-related cell adhesion molecule 1 (CEACAM1) is a member of the immunoglobulin super family of glycoproteins [1,2]. It is expressed on the surface of epithelial and endothelial cells, as well as on cells from the immune system and plays a role in a variety of cellular processes like cell-cell adhesion, proliferation and differentiation, apoptosis and immune response. Several studies have reported down-regulation of CEACAM1 expression in cancers of epithelial origin, including colon [3], breast [4], liver [5], gastric [6] and prostate [7]. The degree of CEACAM1 down-regulation varies between different tissues: in colon cancer the protein is almost completely absent (90% down-regulation), while in breast cancer only about 30% of tumors exhibit a decrease in CEACAM1 expression. Importantly, forced over-expression of CEACAM1 in prostate, breast, colon or liver cell lines results in a decrease of the tumorigenic potential [8-11]. In addition to the widespread CEACAM1 down-regulation, elevated CEACAM1 expression has been observed in lung cancer [12] and malignant melanoma [13,14], underlying the importance of studying the mechanisms which determine CEACAM1 expression.

Several transcription factors function in inducing CEACAM1 transcription. We have previously reported that CEACAM1 transcription can be induced by interferon (IFN) γ [15] through activation of interferon regulatory factor 1 (IRF1), which binds to an interferon response element (ISRE) at the *CEACAM1 *promoter [16]. By performing *in vivo *footprinting with ligation-mediated (LM)-PCR and gel shift assays, we have identified SP1, USF and IRF1 as factors which activate CEACAM1 transcription in HeLa cells and colon cells. An earlier study of the *CEACAM1 *promoter in colon and hepatoma cells implicates USF and possibly HNF-4 and AP-2 in transactivation [17]. More recently, *CEACAM1 *has been identified as a direct transcriptional target of SOX9 in colon cells, by a variety of methods including microarrays, analysis of SOX9 deficient mice, and chromatin immunoprecipitation (ChIP) [18]. While the above-mentioned studies have addressed mainly the mechanisms of activation of the *CEACAM1 *promoter, a single study has addressed the down-regulation of CEACAM1, by implicating the SP2 transcription factor as a direct repressor of CEACAM1 transcription in rat prostate cells [19].

In this work, we have focused on the analysis of the *CEACAM1 *promoter in breast cancer cell lines that vary in CEACAM1 mRNA expression from none (MCF7), to moderate (MDA-MB-468), to higher levels (MCF10A) approximating those found in normal breast. MCF7 cells have played an important role in our 3D model of mammary morphogenesis, where CEACAM1- deficient MCF7 cells fail to form glands with lumena, while forced expression of CEACAM1 restores lumen formation [20]. In contrast, MCF10A cells that express CEACAM1 mRNA in levels similar to normal breast epithelia, form abundant glands in 3D culture [21]. When CEACAM1 was silenced by antisense in the related MCF10F cell line, these cells failed to form glands with lumena [22]. Given that these two cell lines (MCF7 and MCF10A) vary dramatically in their mRNA expression of CEACAM1 with important biological consequences in terms of phenotypes, they were chosen for promoter analysis studies. The choice of MDA-MB-468 as a cell line with intermediate expression of CEACAM1 was prompted by its response to IRF-1 leading to a reduction of survivin expression and a return to a more normal breast epithelial phenotype [23]. In this respect, we predict that the change in phenotype may also be dependent on CEACAM1 expression.

We have studied the *CEACAM1 *promoter activation in these 3 breast epithelial cell lines by performing *in vivo *footprinting using LM-PCR. We have identified protected binding sites at the *CEACAM1 *promoter that correspond to the footprints for SP1, USF and IRF1 identified in our earlier study in colon cells [16]. We have confirmed the binding of these transcription factors to the promoter region by chromatin immunoprecipitation (ChIP) and have detected binding of USF factors even in the absence of CEACAM1 transcription in MCF7 cells. In two out of three of the breast cell lines studied (MCF10A and MDA-MB-468) IRF1can be detected at the ISRE before induction with IFN-γ, together and possibly in competition with IRF2, which can function to modulate CEACAM1 expression level. Silencing of IRF1 and USF1 but not USF2 by RNAi resulted in a significant decrease in CEACAM1 protein expression in MDA-MD468 cells. The inactive *CEACAM1 *promoter in MCF7 cells displays a partially open chromatin structure with significant histone hypoacetylation, which could play a role in the promoter down-regulation.

## Methods

### Cell culture, reagents, and treatments

MCF7 and MDA-MB-468 cells were grown in a 5% CO_2 _incubator at 37°C in MEM supplemented with 1% Sodium Pyruvate, 0.15% Sodium Bicarbonate, 1 × Non-essential Amino Acids, 1 × Penicillin/Streptomycin/Amphotericin B and 10% heat-inactivated FBS. MCF10A cells were cultured in DMEM/F-12 (50/50), supplemented with MEGM SingleQuot Kit (Lonza) and 10% heat-inactivated FBS.

For interferon γ mediated induction of CEACAM1, MCF7 cells were seeded at a density of 1.5 × 10^6 ^cells in 6-well plates 24 h prior to treatment. Human recombinant interferon γ (Pierce) was added to the medium at a concentration of 500 U/ml for 6 h. After incubation, RNA and proteins were isolated as described below.

Trichostatin A treatment was performed with MCF7 cells seeded at a density of 0.8 × 10^6 ^cells in 12-well plates. 24 h after seeding the cells, Trichostatin A (Sigma) at a concentration of 1 μM was added for 0, 6 h and 24 h, respectively, together with DMSO controls. RNA from each time point was isolated as described below.

The following antibodies used for chromatin immunoprecipitation and Western blot were from Santa Cruz Biotechnology: anti-SP1 (PEP-2, sc-59), anti-SP2 (K-20, sc643 and H-282, sc-11400), anti-USF1 (C-20, sc-497), anti-USF2 (N-18, sc-861), anti-IRF-1 (C-20, sc-497), anti-IRF-2 (C-19, sc-498). Western blots for CEACAM1 were performed with mAbT84.1 [24] and anti-β-actin antibody was from Abcam. Anti-trimethyl-Histone H3 (Lys27), anti-trimethyl-Histone H3 (Lys9), clone 6F12-H4 and anti-acetyl-Histone H3(Lys9/18) antibodies used for ChIP were from Millipore.

### RNA isolation and RT-PCR

Total RNA was isolated by the RNeasy mini kit (Qiagen). The RNA was treated with RNase-free DNase set (Qiagen), and RNA (2 μg) was reverse-transcribed in a 20 μl reaction using random hexamers and Superscript III (Invitrogen) according to the manufacturer's instructions. 1/20 to 1/100 of the reaction was used for semi-quantitative PCR with gene-specific primers and Phire Hotstart DNA Polymerase (New England Biolabs) for 32-35 cycles. The products were resolved on 2% agarose gels and visualized by staining with SYBR Green I (Invitrogen). Gels were photographed on a GelLogic 200 Imaging System.

For real time PCR, 1/1200 of the reverse transcription reaction was used to quantitate GAPDH mRNA and 1/20 of the reverse transcription reaction was used to measure CEACAM1 mRNA levels. The reactions were performed on iQ5 Multicolor RealTime PCR Detection System (Bio-Rad) in a 25 μl volume with iQ SYBR Green Supermix (Bio-Rad) and primers for CEACAM1 (forward, 5'-TCTACCCTGAACTTTGAAGCCCA; reverse, 5'-TGAGAGACTTGAAATACATCAGCACTG) and anti-GAPDH (forward, 5'-ATCCATGACAACTTTGGTATCGTG and reverse, 5'-ATGACCTTGCCCACAGCCTT). After denaturation for 3 min at 95°C, 40 cycles were performed (10 sec at 95°C, 30 sec at 64°C, 20 sec at 72°C); fluorescence was recorded at the extension step at 72°C. At the end of the reaction melting curves were generated between 55°C and 95°C, for every 0.5°C. CEACAM1 mRNA levels were calculated by using GAPDH for normalisation.

Quantitation of mRNA expression level of CEACAM1, IRF1, IRF2, USF1, USF2, and GAPDH was performed with primers (Table 1, **top**) using the iQ™5 Multicolor Real-Time PCR Detection System (Bio-Rad Laboratories, Hercules, CA). Briefly, 1/50 of cDNA from the reverse transcription reaction was used for qPCR with 20 pmol of each primer in a total volume of 20 μl using the Sense Mix Plus SYBR^® ^(Quantace Inc, Norwood, MA) and the following conditions: initial denaturation step at 94°C for 3 min; followed by 40 cycles of 95°C for 15 sec, 55°C for 15 sec, 72°C for 15 sec. The fluorescence was measured at the end of the extension step at 72°C. Subsequently, a melting curve was recorded between 55°C to 95°C every 0.2°C with a hold every 1 second. Levels of mRNA (triplicate) were compared after correction by use of concurrent GAPDH message amplification.

**Table 1:** Oligonucleotides

qPCR	Forward Primer/Reversed Primer	Size (bp)
EACAM1	5'-TGCTGGCATTGTGATTGGAG-3'	
	5'-ACATGCCAGGGCTACTGCTATC-3'	61
IRF1	5'-CTCTGAAGCTACAACAGATGAGG-3'	
	5'-CTGTAGACTCAGCCCAATATCCC-3'	215
IRF2	5'-GAGTATGCGGTCCTGACTTCAAC-3'	
	5'-CATCGCTGGGCACACTATCAGTCG-3'	259
USF1	5'-TCGTGCAGCTCTCCAAGATAATCC-3'	
	5'-CCTGTTGTCGAAGCACGTCATTG-3'	188
USF2	5'-CAGATGGACAACGAGCTCCTGAG-3'	
	5'-GCATGTGTCCCTCTCTGTGCTAAG-3'	207
GAPDH	5'-CATTGCCCTCAACGACCACTTTGT-3'	
	5'-CACCCTGTTGCTGTAGCCAAATTC-3'	73
		
RNAi	Sequence	
		
IRF1 #1	5'-CCCUGGCUAGAGAUGCAGAUUAAUU-3'	
IRF1 #2	5'-GGGACAUCAACAAGGAUGCCUGUUU-3'	
IRF1 #3	5'-CGGACAGCACCAGUGAUCUGUACAA-3'	
		
IRF2 #1	5'-GGCUUAGUAAUGGAGUAAGUGAUCU-3'	
IRF2 #2	5'-UCUCCUGAGUAUGCGGUCCUGACUU-3'	
IRF2 #3	5'-GAGGAGCAGAUAAACUCCAACACGA-3'	
		
USF1 #1	5'-CCUGGCACUGGUCAAUUCUUUGUGA-3'	
USF1 #2	5'-UGGAUCGUGCAGCUCUCCAAGAUAA-3'	
USF1 #3	5'-CAUCAGUGGCUACCCUGCCACUCAA-3'	
		
USF2 #1	5'-CCAGCGUCCAGUGUGGGAGAUACUA-3'	
USF2 #2	5'-CCAGGAUGUGCUUCAGACAGGAACA-3'	
USF2 #3	5'-GGAUCCUGUCCAAGGCCUGCGAUUA-3'	

### Protein isolation and Western Blot

For total protein extraction, cells at a confluency of about 90% were incubated in RIPA buffer (10 mM sodium phosphate (pH 7.2), 150 mM NaCl, 1% Nonidet P40, 1% Na deoxycholate, 0.1% SDS, 2 mM EDTA, 1 mM DTT), supplemented with 1 mM PMSF, 100 U/ml benzonase, proteinase inhibitor cocktail (Roche) and phosphatase inhibitor cocktail (Pierce). Cells were incubated on ice for 30 min and the lysate was cleared by centrifugation and kept at -80°C [25]. Typically 25-50 μg of protein from the lysate was loaded on 4-12% polyacrylamide SDS gel (Novex) and the proteins were transferred from the gel to PVDF membrane. The Western blot was performed with infrared dye labelled secondary antibodies (Li-COR Biosciences) and signal was detected on the Odyssey Infrared Imaging System (LI-COR Biosciences).

### *In vivo *footprinting with dimethyl sulfate

MDA-MB-468, MCF7 or MCF10A cells (4-6 × 10^6^) at a confluency of about 90% were treated with 0.1% dimethyl sulfate (DMS, Sigma) for 5 min at room temperature. After three washes with PBS, DNA was isolated with DNeasy Tissue kit (Qiagen), eluted in TE pH 7.5 and stored at 4°C. Purified genomic DNA isolated from MDA-MB-468 cells was incubated with 0.5% DMS for 2 min at room temperature and then treated with piperidine as described in [26]. G and G+A Maxam-Gilbert sequencing reactions with purified genomic DNA (from MDA-MB-468 cells) were performed according to Pfeifer et al. [26]. *In vivo *footprinting with LM-PCR was performed essentially according to [27], with the use of an infrared-labeled primer and subsequent detection on a LI-COR DNA sequencer [28]. The primer sets for the coding strand were:

P1: 5'-GTTGCAAAGAAAATAATTACCAC, biotinylated at 5' end

P2: 5'-CCACATTTGGATATGCCAGGGTTCTC

P3: 5'-TGGATATGCCAGGGTTCTCTGTGTGCTGC, labelled with IR700 (LI-COR Biosciences). The primer set for the noncoding strand were:

P1AS: 5'-TCCTGCTGGCCCTGTCTTCAC, biotinylated at 5' end

P2AS: 5'-TTCACCTGTGGAGGAGAGCTTGGGC

P3AS: 5'-GAGAGCTTGGGCTCCAGGAACGCTTCGAG, labelled with IR700.

### Chromatin Immunoprecipitation

MDA-MB-468, MCF7 or MCF10A cells (4-6 × 10^6^) at a density of 90% were crosslinked with 1% formaldehyde (Sigma) for 10 min at room temperature. After washing with PBS, cells were lysed in 750 μl buffer containing 1% SDS, 10 mM EDTA, 50 mM tris-HCl, pH 8.1, for 30 min on ice. Lysates were subjected to sonication on a Branson digital sonifier for 8 × 10 sec at 40% amplitude. These conditions typically sheared DNA to fragments between 200 bp and 1.5 kb in size. The lysates were cleared by centrifugation at 14 000 rpm, 7 min, 4°C, frozen in liquid nitrogen and stored at -80°C until further use. For immunoprecipitation, after preclearing the lysates with Protein G Plus agarose beads (Pierce) for 1 h at 4°C, the beads were removed and the supernatant was diluted 1:10 in buffer containing 0.01% SDS, 1.1% Triton X-100, 1.2 mM EDTA, 16.7 mM Tris-HCl, pH 8.1, 167 mM NaCl. Aliquots of the lysates (200 μl for transcription factors and 100 μL for histone modification) were incubated overnight with 5 μg of antibody at 4°C on a rotating platform, followed by addition of Protein G Plus agarose for 2 h. The beads were washed at 4°C once in low salt buffer (0.1% SDS, 1% Triton X-100, 2 mM EDTA, 20 mM Tris-HCl ph 8.1, 150 mM NaCl), once in high salt buffer (0.1% SDS, 1% Triton X-100, 2 mM EDTA, 20 mM Tris-HCl ph 8.1, 500 mM NaCl), once in LiCl buffer (0.25 mM LiCl, 1% Nonidet P40, 1% Na deoxycholate, 1 mM EDTA, 10 mM Tris pH 8.1) and twice in TE buffer. DNA was eluted with 1% SDS/0.1 M NaHCO_3 _and the crosslinks were reversed by incubation at 65°C for 4 h. The DNA was purified by treatment with Proteinase K (Sigma) for 2 h at 50°C, extracted with phenol/chlorofom/isoamyl alcohol and precipitated with ethanol. DNA was dissolved in Tris-HCl, pH 7.5 and amplified using Phire Hotstart DNA polymerase using the following primers for the *CEACAM1 *promoter region: forward, 5'-GGTCTGGGAAACCAAAATGTAGACAG, reverse 5'-CTCTGTGCTGAGCCTCCTCCCT. Aliquots of the reactions were resolved on 2% agarose gel containing SYBR Green I and visualized on a GelLogic 200 Imaging System.

### RNAi treatment

Cells were transfected with RNAi oligos (Table 1, bottom) using the Lipofectamine™ RNAiMAX transfection agent. Cells were split, seeded at 50% confluence in T25 flasks overnight, washed twice with 1× PBS, and treated with RNAi oligos (200 nM) and tranfection agent (1:100 dilution) in Opti-MEM^® ^I reduced serum medium for 18 hours without removing the transfection solution, after which time the cells were supplemented MEM containing 10% fetal bovine. Cells were treated with RNAi oligos for a total of 72 hours.

## Results

### CEACAM1 mRNA expression in MDA-MB-468, MCF10A and MCF7 cells

**Figure 1:**
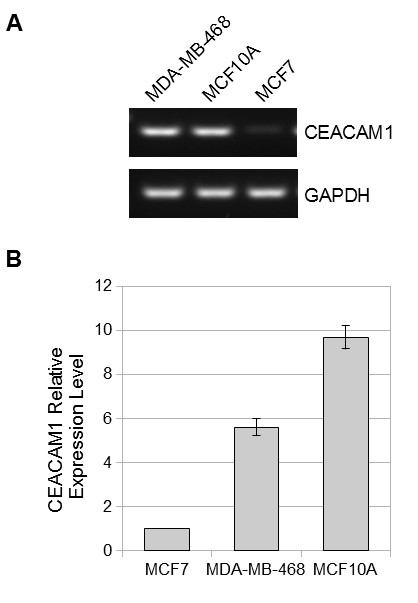
**CEACAM1 mRNA expression in MDA-MB-468, MCF10A and MCF7 cells**. A. RT-PCR with RNA isolated from MDA-MB-468, MCF10A and MCF7 cells and amplified with primers detecting all isoforms of CEACAM1. GAPDH has been used as loading control. B. CEACAM1 mRNA levels in the three cell lines were quantified by real time PCR, using GAPDH for normalisation.

To study the factors responsible for CEACAM1 transcription in breast epithelilal cells, we chose three well studied cell lines that vary in their mRNA expression levels of CEACAM1 from none (MCF7) to moderate (MDA-MB-468) to high (MCF10A). To assay the CEACAM1 mRNA levels in these cell lines, we isolated RNA and performed RT-PCR using a primer pair that detects all CEACAM1 splice variants (Figure 1A). The mRNA in the three cell lines was quantified by real-time PCR using GAPDH for normalization (Figure 1B). As expected, MCF10A and MDA-MB-468 cells expressed CEACAM1 mRNA with MCF10A levels greater than MDA-MB-468, while in MCF7 cells, the CEACAM1 transcript was very low.

### *In vivo *DMS footprinting of the *CEACAM1 *promoter

**Figure 2:**
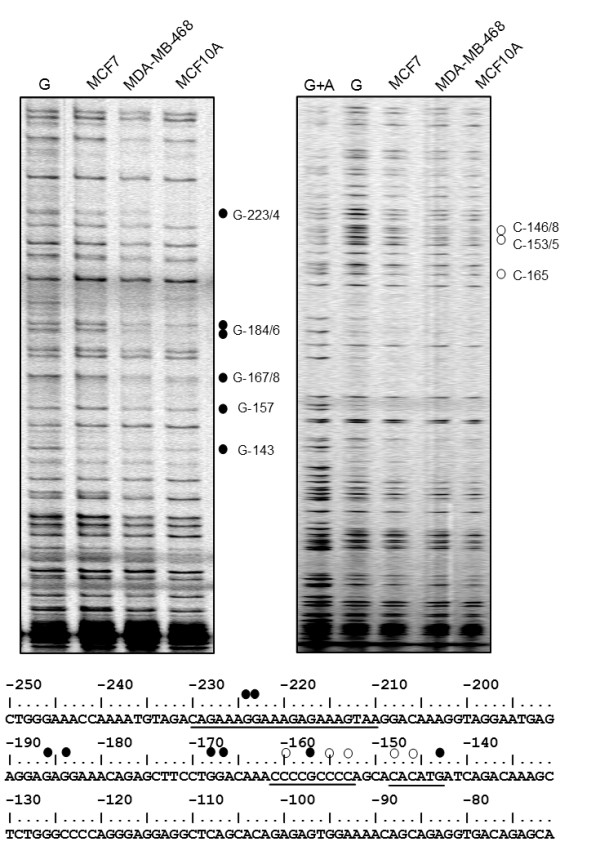
***CEACAM1 *promoter regions protected through binding of transcription factors**. Top, *In vivo *footprinting with LM-PCR performed with DNA isolated from DMS-treated MDA-MB-468, MCF10A and MCF7 cells. Top left, footprinting on the coding DNA strand; top right, footprinting on the non-coding DNA strand. G+A, a Maxam-Gilbert sequencing reaction performed with DNA isolated from MDA-MB-468 cells. G, purified genomic DNA from MDA-MB-468 cells modified with DMS *in vitro *and subjected to LM-PCR. Closed circle, protected bases on the coding strand; open circle, protected bases on the non-coding strand. Bottom, part of the sequence of the proximal *CEACAM1 *promoter, from -70 bp to -250 bp. Underlined are previously identified binding sites for USF, -143-148, SP1, -153-162, and IRF1, -210-230. Closed and open circles show the protected bases identified by the *in vivo *footprinting experiment.

We next proceeded to determine transcription factor binding sites on the *CEACAM1 *promoter, by performing *in vivo *footprinting mediated by LM-PCR. Since we were interested in comparing the *CEACAM1 *promoter occupancy in breast cells to published data for the CEACAM1 promoter in colon cells [16], we chose to look at the immediate promoter sequence, between -65 to -365 bp. We treated MCF7, MDA-MB-468 and MCF10A cells with dimethyl sulfate (DMS) *in vivo*, isolated DNA and subjected it to LM-PCR. As a control for band intensity we used purified genomic DNA isolated from MDA-MB-468 cells, digested *in vitro *with DMS. DNA from MDA-MB-468 cells was also subjected to Maxam-Gilbert sequencing and used as a marker. Using primers that amplify the coding DNA strand, we were able to distinguish several protected bases on DNA form MDA-MB-468 and MCF10A cells, with both cell lines giving very similar patterns (Figure 2, top left). The G at -143 maps in a site for USF1/2, while G at -157 marks a site for binding of SP1. The double band at -223/4 is part of a binding site for IRF-1. All three binding sites were also protected in colon cells, as reported previously [16]. In breast cells, we have detected two additional footprints, at G -167-168 and G -184-186. For MCF7 cells, we have failed to detect any protected bases, except for G-143, in the binding site for USF1/2. Footprinting with primers amplifying the antisense DNA strand confirmed the SP1 and USF1/2 binding sites in MDA-MB-468 and MCF10A cells, as well as the detection of USF1/2 on the promoter in MCF7 cells (Figure 2 top right).

### Direct binding of SP1, USF1/2 and IRF1 at the *CEACAM1 *promoter assayed by ChIP

**Figure 3:**
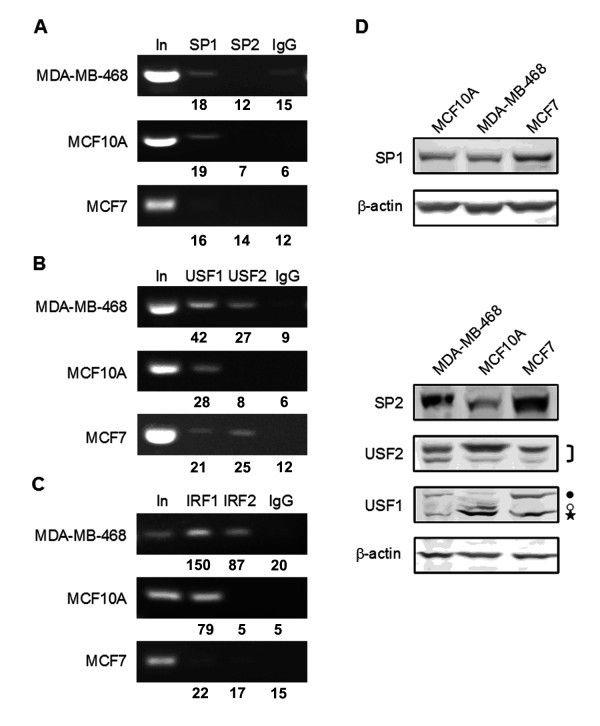
**Direct binding of transcription factors to the *CEACAM1 *promoter region**. Chromatin immunoprecipitations were performed with antibodies to SP1 and SP2 (K-20) (**A**), USF1 and USF2 (**B**), and IRF1 and IRF2 (**C**) in MDA-MB-468, MCF10A and MCF7 cells. Primers amplify the *CEACAM1 *promoter region. In: input DNA from the ChIPs; IgG, rabbit IgG used as negative control, numbers refer to percent of input. **D**. Total cellular protein lysates from MDA-MB-468, MCF10A and MCF7 cells were subjected to Western blot and probed with antibodies to SP1, SP2 (K-20), USF1 and USF2; β-actin was used as a loading control. Closed circle, acetylated form of USF1, open circle, phosphorylated form of USF1, asterisk, USF1 (see Corre et al., [30])

To confirm that SP1, USF1/2 and IRF1 indeed bind to the promoter sites identified by in vivo footprinting, we performed chromatin immunoprecipitation. ChIP with antibodies to SP1 indicated that SP1 was weakly bound to the *CEACAM1 *promoter in MCF10A and MDA-MB-468 cells (Figure 3A). At the same time, SP1 was very low in MCF7 cells by ChIP, despite being expressed at a higher level compared to MDA-MB-468 and MCF10A (Figure 3D). We also tested the binding of SP2 to the *CEACAM1 *promoter, since SP2 has been reported to bind to the *CEACAM1 *promoter in rat prostate cells as a transcriptional repressor [19]. We were unable to detect binding of SP2 to the human *CEACAM1 *promoter in any of the cell lines tested, despite the fact that the protein was expressed in all three lines (Figure 3D). Similar results were obtained using a different SP2 antibody (H-282; data not shown).

We further performed ChIP with antibodies to USF1 and USF2, which are known to bind to promoter sequences predominantly as a USF1/USF2 dimer [29]. We detected USF1 binding at the promoter region in all three cell lines tested, including MCF7, as predicted by the footprinting data (Figure 3B). We noted that USF1 gives a stronger signal in MDA-MB-468 and MCF10A cells compared to MCF7 cells. USF2 was absent in MCF10A cells where the highest expression of CEACAM1 was observed. Western blot analysis indicated that both USF1 and USF2 were present in all three cell lines but differed in the expression of their molecular sizes (Figure 3D). USF1 has a major molecular species, detectable in all three cell lines (Figure 3D, asterisk), as well as a threonine-phosphorylated molecular species (Figure 3D, open circle) and an acetylated molecular species (Figure 3D, closed circle) reported to exhibit differential transcription potentials [30]. MCF10A cells exhibited detectable levels of the phosphorylated USF1 isoform, while the acetylated USF1 isoform was predominantly expressed in MDA-MB-468 and MCF7 cells.

**Figure 4:**
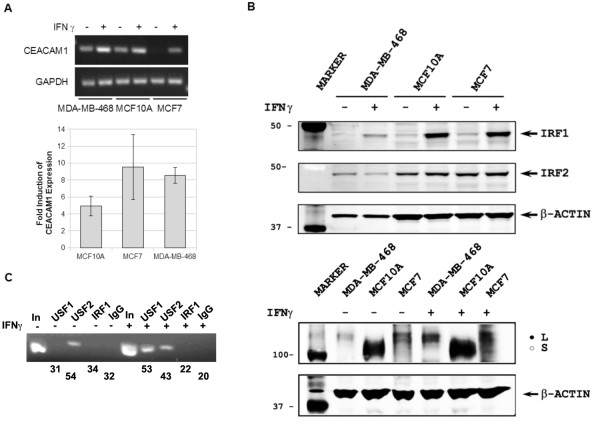
**Induction of CEACAM1 expression in MCF7 cells with IFN γ**. **A**. Top, RT-PCR with total RNA isolated from MCF7 cells, either untreated (-) or treated for 6 h with 500 U/ml of IFN γ (+). Bottom, CEACAM1 mRNA levels were monitored by real time PCR and normalized to GAPDH (triplicates ± SD). **B**. Total cellular protein lysates from MDA-MB-468, MCF10A and MCF7 cells either untreated (-) or treated (+) with 500 u/ml IFN γ were subjected to Western blot and probed with antibodies to IRF1, IRF2, and CEACAM1. β-actin was used as a loading control. Closed and open circle indicate two CEACAM1 isoforms with a different migration from MDA-MB-468 and MCF10A cells, respectively. **C**. Chromatin immunoprecipitation of *CEACAM1 *promoter DNA from MCF7 cells untreated (-) or treated with 500 u/ml of IFN γ for 6 h. Antibodies to USF1, USF2, IRF1 and a control IgG were used, numbers refer to percent of input.

To verify binding of a transcription factor at the IRF-1 binding site, we performed ChIP with an antibody to IRF-1, as well as an antibody to IRF-2. IRF-2 is a well-studied repressor recognizing consensus sites common to the IRF group of proteins [31], thus making it a candidate for modulation of CEACAM1 expression, perhaps opposing IRF-1. While IRF1 binding was evident in MCF10A and MDA-MB-468 cells, there was a very low IRF-1 ChIP signal in MCF7 cells (Figure 3C). On the other hand, strong IRF-2 binding to the *CEACAM1 *promoter was detected only in the MDA-MB-468 cells. Western blot analysis demonstrated that IRF2 is expressed in both MCF10A and MCF7 cells, but weakly in MDA-MB-468 cells (Figure 4B). Our data is consistent with the footprinting results that show no IRF1 binding at the ISRE site in MCF-7 cells. The possible role for IRF-2 as a transcriptional repressor is unlikely since it was detected only in the ChIP analysis on MDA-MB-468 cells that are able to express CEACAM1.

### Interferon γ induction of CEACAM1

We next induced CEACAM1 expression by treating the cells with interferon (IFN) γ and looked for changes in the transcription factor binding to the *CEACAM1 *promoter in MCF7 cells by ChIP. Previously, we demonstrated that treatment with IFN γ induces CEACAM1 in colon cells through induction of IRF-1 [16] and thus reasoned that IFN γ might have a similar effect on the CEACAM1 transcription in breast epithelial cells. We treated MDA-MB-468, MCF10A and MCF7 cells with IFN γ under the conditions described for colon cells (500 U/mL for 6 h) and isolated RNA and protein to monitor CEACAM1 induction. RT-PCR demonstrated that IFN γ treatment indeed up-regulated CEACAM1 mRNA in all three cell lines tested (Figure 4A). Although CEACAM1 transcription was induced several fold in MCF7 cells, the steady-state mRNA level in these cells did not reach the CEACAM1 mRNA levels in uninduced MDA-MB-468 and MCF10A cells. We also detected a robust induction of IRF-1 (Figure 4B) by Western blot analysis, consistent with the mechanism of IFN γ induction described for colon cells. On the other hand, there was no change in IRF-2 levels (Figure 4B), in agreement with a previous report [32]. IFN γ treatment also induced CEACAM1 in MDA-MB-468 and MCF10A cells, but in MCF7 cells CEACAM1 was still undetectable.

Since CEACAM1 was not induced in IFN-γ treated MCF7 cells, we performed additional ChIP analysis in these cells. As expected, we detected USF2 at the promoter both before and after induction (Figure 4C). The USF1 signal increased considerably after IFN γ treatment, suggesting that the level and/or strength of USF1 binding at the promoter might influence CEACAM1 transcription. We could not detect binding of IRF1 to the promoter region, likely reflecting the low level of CEACAM1 induction.

### Chromatin structure at the *CEACAM1 *promoter in MDA-MB-468, MCF10A and MCF7 cells

**Figure 5:**
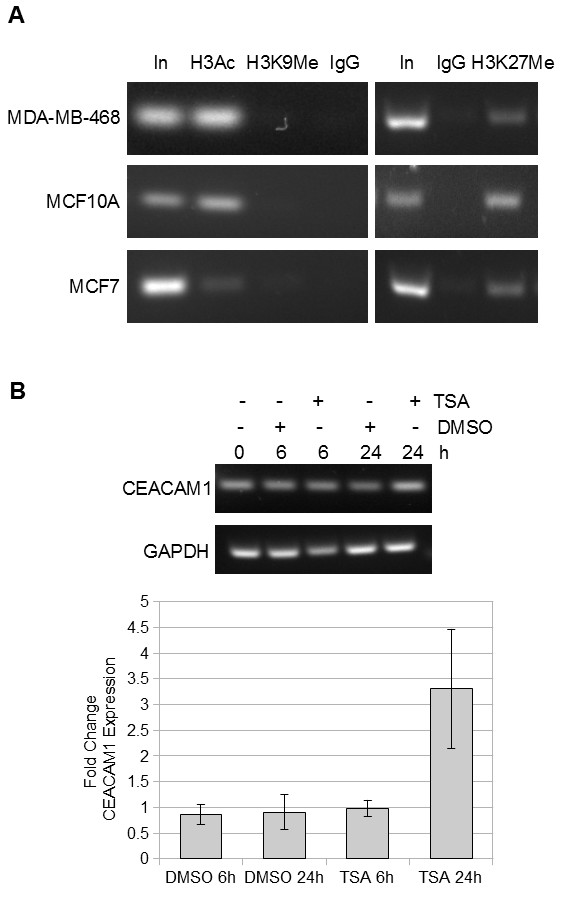
**Chromatin structure at the *CEACAM1 *promoter in MDA-MB-468, MCF10A and MCF7 cells**. **A**. Chromatin immunoprecipitations of *CEACAM1 *promoter DNA from MDA-MB-468, MCF10A and MCF7 cells with antibodies detecting acetylated Lys9 and Lys 18 of histone H3 (H3Ac), trimethylated Lys 9 of histone H3 (H3KMe), and trimethylated Lys 27 of histone H3 (H3K27Me). IgG was used as negative control; In: input DNA. **B**. MCF7 cells were treated with either DMSO or Trichostatin A for 0 h, 6 h, and 24 h, respectively, and total RNA was isolated and subjected to RT-PCR with primers amplifying CEACAM1 (top). GAPDH was used as a loading control. Bottom, CEACAM1 mRNA levels were quantified by real time PCR, using GAPDH for normalization.

In order to determine whether chromatin structure plays a role in modulating CEACAM1 transcription, we monitored the promoter region for histone modifications. First, we used an antibody which recognizes acetylated lysine 9 and lysine 18 of histone H3, marks associated with actively transcribed genes, and probed the *CEACAM1 *promoter by ChIP in MDA-MB-468, MCF10A and MCF7 cells. While both MDA-MB-468 and MCF10A cells exhibited a strong signal for acetylated histone H3, in MCF7 cells the *CEACAM1 *promoter showed significantly decreased acetylation, in agreement with the CEACAM1 expression pattern in these cell lines (Figure 5A). Since a hypoacetylated promoter can be activated by histone deacetylase inhibitors, we treated MCF7 cells with 1 μM Trichostatin A for 0 h, 6 h, and 24 h, respectively and monitored CEACAM1 mRNA levels by RT-PCR. Trichostatin A treatment induced a modest increase in CEACAM1 mRNA levels (Figure 5B), suggesting that apart from reduced acetylation there are other factors contributing to CEACAM1 down-regulation. We next performed ChIP with antibodies to trimethyl-histone H3 Lys 9, a well studied histone modification linked to condensed chromatin structure and gene silencing [33]. Neither MDA-MB-468 nor MCF10A cells showed H3 Lys9 trimethylation at the *CEACAM1 *promoter; for MCF7 cells the signal was also essentially negative. We also performed ChIP to detect histone H3 lysine 27 trimethylation at the *CEACAM1 *promoter, another mark of silenced chromatin [34,35]. Unexpectedly, all three cell lines exhibited strong H3K27 trimethylation at the *CEACAM1 *promoter region (Figure 5A). Thus, it is unlikely that the role of the H3K27 mark on the *CEACAM1 *promoter is solely down-regulation of gene expression. It is also unlikely that H3K27 trimethylation is responsible for CEACAM1 down-regulation in MCF7 cells.

### Effect of RNAi for transcription factors on CEACAM1 expression in MDA-MB-468 cells

**Figure 6:**
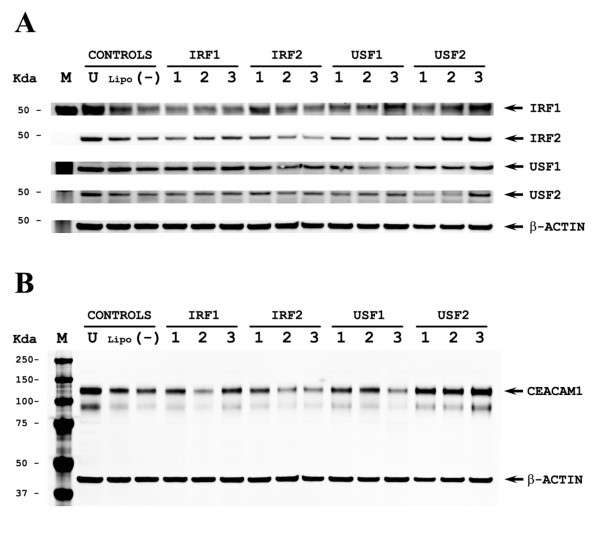
**Effect of RNAi silencing of IRF1, IRF2, USF1 and USF2 on CEACAM1 expression in MDA-MB-468 cells**. **A**. MDA-MB-468 cells were treated with the following controls: U (untreated), lipofectamine (no RNAi), or non-specific RNAi (-) and IRF1, IRF2, USF1, and USF2 levels measured by western blot analysis after 72 hrs. Cells treated with RNAi for IRF1 (3 oligos), IRF2 (3 oligos), USF1 (3 oligos), or USF2 (2 oligos) were analyzed for IRF1, IRF2, USF1, and USF2 expression by western blot analysis after 72 hrs. Lysates were probed with antibody to β-actin to ensure equal protein loading in each lane. **B**. MDA-MB-468 cells were treated with the following controls: U (untreated), lipofectamine (no RNAi), or non-specific RNAi (-) and CEACAM-1 levels measured by western blot analysis after 72 hrs. Cells treated with RNAi for IRF1 (3 oligos), IRF2 (3 oligos), USF1 (3 oligos), or USF2 (2 oligos) were analyzed for CEACAM1 expression by western blot analysis after 72 hrs. Lysates were probed with antibody to β-actin to ensure equal protein loading in each lane.

We conclude from the above analyses that IRF1 and USF1 are critical transcription factors in the regulation of CEACAM1 in the breast cell lines analyzed. Since the MDA-MB-468 cells have intermediate levels of CEACAM1 mRNA expression, lower than MCF-10A and higher than MCF7 cells (Figure 1), we predicted that they will be most sensitive to alterations in the levels of these critical transcription factors. In order to test this prediction, we transfected these cells with RNAi oligos to IRF1 and USF1 plus RNAi to the related transcription factors IRF2 and USF2 that bind to the analogous sites in the *CEACAM1 *promoter. Several RNAi oligos plus non-specific RNAi were tested to confirm the ability of RNAi to silence their specific targets at mRNA (Table 2) and the protein level (Figure 6A). Compared to the controls that included no treatment, lipofectamine only, or unspecific RNAi, we found a dramatic down-regulation of CEACAM1 protein expression by RNAi to IRF1, IRF2, and USF1, but not to USF2 (Figure 6B). These results confirm our prediction that IRF1 and USF1 critically regulate the expression of CEACAM1, and further, add a role for IRF2. This is especially interesting since in other systems, IRF-2 has been shown to antagonize IRF1 [36]. The implications of this finding will be discussed later.

**Table 2:** mRNA levels of CEACAM1 after silencing of IRF-1, IRF-2, USF1 and USF2

Treatment	Relative amount^1^	Percent of control^2^
None	1.4 ± 0.1	93
Lipofectamine	1.4 ±0.1	93
Negative control^3^	1.5 ±0.1	100
		
IRF1 #1	0.9 ±0.1 *	60
IRF1 #2	0.8 ±0.2 *	53
IRF1 #3	1.3 ±0.1	87
		
IRF2 #1	1.4 ±0.1	93
IRF2 #2	1.0 ±0.2 *	67
IRF2 #3	0.9 ±0.1 *	60
		
USF1 #1	1.1 ±0.2 *	73
USF1 #2	1.1 ±0.2 *	73
USF1 #3	1.0 ±0.1 *	67
		
USF2 #1	0.9 ±0.1 *	60
USF2 #2	0.9 ±0.1 *	60
USF2 #3	1.0 ±0.2 *	67

^1^Relative to GAPDH. assayed in triplicate, ±SD, * indicates p < 0.01 compared to negative control.

^2^Compared to negative control,

^3^RNAi medium GC control from Invitrogen.

## Discussion

We have studied the *CEACAM1 *promoter region in three breast epithelial cell lines, that vary in CEACAM1 mRNA expression from none (MCF7) to intermediate (MDA-MB-468) to high (MCF10A). We have performed *in vivo *footprinting with DMS on the C*EACAM1 *promoter region and have detected several protected sites, indicating binding of multiple transcription factors to the promoter. These binding sites correspond well with previous footprinting data for the *CEACAM1 *promoter in colon cells [16], with some differences. As in colon cells, the breast epithelial cells expressing CEACAM1 exhibit footprints at the binding sites for SP1, USF1, USF2 and the interferon response element, suggesting a common regulation mechanism for these cells. However, we were able to detect protein/DNA interactions at the interferon response element even before induction with IFN γ. This result indicates that perhaps even small quantities of IRF1 bound to the promoter may function in transcriptional activation of the *CEACAM1 *promoter. We have also observed two new protected sites at the *CEACAM1 *promoter in breast cells. The first one, around nt -165-168, has a weak consensus binding site for NFkB, but we could not confirm binding of NFkB by ChIP to the *CEACAM1 *promoter (data not shown). The second one, around nucleotides -184-186 remains to be investigated.

USF1 and USF2 have emerged as key regulators of CEACAM1 transcription. While USF binding to the *CEACAM1 *promoter has been observed previously, we have extended our understanding of USF function in CEACAM1 transcription by demonstrating that USF proteins remain bound to the promoter in it's inactive state, by both *in vivo *footprinting and ChIP. We have also observed weaker binding of USF1 compared to USF2 in MCF7 cells that do not express CEACAM1, and an increase in USF1 binding to the *CEACAM1 *promoter after IFN γ activation. While ubiquitously expressed in mammalian cells [37], the ratio of USF1 to USF2 protein varies in different cell lines and in different phases of the cell cycle, indicating that the USF proteins are subject to extensive regulation [38,39]. It has recently been demonstrated that under mild stress conditions USF1 can undergo threonine phosphorylation that increases the protein's activation potential [30]. In addition, the same study documents that under acute stress or viral infection USF1 undergoes phosphorylation-dependent acetylation, a modification which negatively affects transcription. We have detected a protein band on Western blots corresponding to the phosphorylated form of USF1 in MCF10A cells, which express the highest amount of CEACAM1 mRNA, but not in MDA-MB-468 cells or MCF7 cells. At the same time, in our analysis both MCF7 cells and MDA-MB-468 cells express a protein corresponding to the phospho-acetylated form of USF1, which could play a role in downregulating transcription at the *CEACAM1 *promoter. Our data is also broadly consistent with a report that in breast cancer cells the USF proteins have altered transcription activation potential compared to the nontumorigenic MCF10A cells, despite being expressed at similar levels [38]. Of particular interest is a report that USF1 interacts with both SET7/9, a histone methyltransferase, and with pCAF, a histone H3 acetyltransferase, that implicates USF1 in recruiting histone modifying enzymes to promote transcriptional activation and maintain open chromatin structure [40]. In this light our finding that the *CEACAM1 *promoter exhibits a significant decrease in histone acetylation in MCF7 cells might reflect a suboptimal presence of USF1 at the promoter in this cell line. In our assay the inactive *CEACAM1 *promoter in MCF7 cells doesn't appear to differ in two key histone modification compared to MCF10A and MDA-MB-468 cells, tri-methylation of H3 Lys 9 and tri-methylation of H3 Lys 27. This result, together with the detection of USF proteins at the inactive *CEACAM1 *promoter, suggests that the chromatin structure at the promoter might be partially open, possibly facilitating upregulation of the gene under specific conditions.

We were particularly interested in identifying proteins acting as repressors of CEACAM1 transcription, since CEACAM1 mRNA levels are downregulated in many cancer types. Since a published report has identified SP2 as a direct repressor of CEACAM1 transcription in rat prostate cells [19], we tested SP2 binding to the *CEACAM1 *promoter by ChIP in MDA-MB-468, MCF10A and MCF7 cell lines. We used two different antibodies, both of which indicated a similar expression of SP2 in the three cell lines, but we were unable to immunoprecipitate *CEACAM1 *promoter DNA. The proposed SP2 binding site in rat prostate cells overlaps with the SP1 site on the human *CEACAM1 *promoter. Thus, assuming a similar mechanism between rat and human, SP2 would compete for binding with SP1. However, it has been reported that SP1 and SP2 have different DNA binding preferences [41], which make binding of the two proteins to the same site unlikely. The fact that we do not detect a footprint in MCF7 cells in that region additionaly argues against involvment of SP2 as a repressor stably bound to the human *CEACAM1 *promoter. However, we can not exclude the possibility that there are differences between prostate and breast cells in CEACAM1 expression; the discrepancy might also indicate a difference between rat and human cells.

Another transcription factor that could act as repressor of CEACAM1 transcription is IRF2 [42]. IRF2 recognizes the same consensus sequence as IRF1 [43,44] and generally opposes the function of IRF1, leading to downregulation of target genes [36]. We were able to detect IRF2 in two of the cell lines we studied, MDA-MB-468 and MCF7, but IRF2 was largely absent from MCF10A cells in which the highest expression of CEACAM1 mRNA is observed. This pattern of expression is consistent with reports that IRF2 expression level increases with cancer progression [45]. In agreement with the expression pattern, we were able to immunoprecipitate the *CEACAM1 *promoter region with antibodies to IRF2 in MDA-MB-468 cells, but not in MCF10A cells. This result suggests that the ratio between IRF1 and IRF2 in a given cell might modulate the level of CEACAM1 expression, as has been demonstrated for other target genes regulated by IRF1 and IRF2 [46]. In MCF7 cells, in which the *CEACAM1 *promoter is in an inactive state, we do not detect binding of either IRF1 or IRF2, suggesting that if IRF2 contributes to CEACAM1 down-regulation, it is not required to stably bind to the DNA to maintain the inactive state.

Since our results predict that USF1 and IRF1 are critical regulators of CEACAM1 expression in breast epithelial cells, we further predicted that down-regulation of these two transcription factors would reduce CEACAM1 expression. We chose the MDA-MB468 cell line to test this prediction because it had relatively high expression of CEACAM1 at both the mRNA and protein level. In contrast, MCF10A cells had high levels of CEACAM1 expression at the mRNA, making it a good cell line for transcriptional regulation, but a poor cell line for testing protein expression. As predicted, silencing either USF1 or IRF1 by RNAi significantly reduced expression of CEACAM1 at the protein level. A further prediction was that USF2 and perhaps IRF2 would have no or little effect on expression. Although this was indeed the case for RNAi to USF2, surprisingly, RNAi to IRF2 had the same effect as IRF1. This latter result can be explained in terms of the varied reports on the role of IRF2 as both a repressor and activator of genes [31]. In the case of gene activation, IRF2 has been shown to positively regulate vascular cell adhesion molecule-1 in muscle [47], to up-regulate IL-7 production in human intestinal epithelial cells [48], to activate HPV-16 E6-E7 promoter in keratinocytes [49], and to be required for CIITA type IV promoter activation [50]. Furthermore, in a transfection assay, IRF2 was required for NFκB translocation to the nucleus and subsequent activation of TNFα transcription [51]. This latter finding is especially intriguing since NFκB activation has been linked to IFN-γ/CEACAM1 mediated effects in Neisseria menningitidis invasion of epithelial cells [52], and we have identified a putative NFκB binding site in the *CEACAM1 *promoter. However, further work is required to determine if and under what circumstances this binding site becomes operational. NFκB is a central mediator of inflammation and it has been shown that IRF2 regulates the inflammatory and apoptotic response of mice to LPS [53]. Furthermore, mice deficient in IRF2 have a defect in the production of T_H_1 helper T-cells and NK cells [54], thus linking IRF2 to the production of a pro-inflammatory response.

Besides a putative NFkB binding site in the *CEACAM1 *promoter, we have identified a putative RUNX1 binding site that is of potential interest because of the role of this transcription factor in granulopoiesis [55] and the finding that CEACAM1 is a marker of granulocyte activation [56,57]. Taken together, these data may indicate that breast cells may respond to inflammation by up-regulation of CEACAM1. However, subsequent events, perhaps chronic exposure to inflammatory cells/cytokines, may lead to down-regulation of CEACAM1, thus accounting for the over-all decrease in CEACAM1 observed in breast cancer. Future studies will be aimed at studying the effects of chronic inflammation on the *CEACAM1 *promoter.

In summary, we have identified USF1 and IRF1 as critical regulators of CEACAM1 expression in breast cells by combined in vivo footprint and ChIP analysis and shown that treatment with IFN-γ up regulates both USF1 and IRF1 binding to the *CEACAM1 *promoter followed by increased protein expression of CEACAM1. Down-regulation of these two transcription factors by RNAi significantly reduces the expression of CEACAM1 in MDA-MB-468 cells. These studies suggest that CEACAM1 is involved in the response of breast cells to inflammation.

## Authors' contributions

Experiments and data analysis were performed by MG, CJC and TN. The manuscript was written by MG and edited by JES. All authors read and approved the final manuscript.
